# Selenium in Radiation Oncology—15 Years of Experiences in Germany †

**DOI:** 10.3390/nu10040483

**Published:** 2018-04-13

**Authors:** Ralph Muecke, Oliver Micke, Lutz Schomburg, Jens Buentzel, Klaus Kisters, Irenaeus A. Adamietz, on behalf of AKTE

**Affiliations:** 1 Radiotherapy RheinMainNahe, 55543 Bad Kreuznach, Germany; 2 Department of Radiotherapy and Radiation Oncology, Marien Hospital Herne, Ruhr University Bochum, 44801 Bochum, Germany; irenaeus.adamietz@elisabethgruppe.de; 3 Department of Radiotherapy and Radiation Oncology, Franziskus Hospital, 33615 Bielefeld, Germany; strahlenklinik@web.de; 4 Institute for Experimental Endocrinology, Charité Berlin, 10117 Berlin, Germany; lutz.schomburg@charite.de; 5 Department of Otolaryngology, Südharz Hospital Nordhausen, 99734 Nordhausen, Germany; Jens.Buentzel@shk-ndh.de; 6 Department of Internal Medicine, St. Anna Hospital, 44649 Herne, Germany; Klaus.Kisters@elisabethgruppe.de

**Keywords:** selenium, Radiation Oncology, selenium measurement, selenium supplementation

## Abstract

**Introduction:** Se measurement and supplementation in radiation oncology is a controversial issue. The German Working Group Trace Elements and Electrolytes in Oncology (AKTE) has conducted a number of studies on this issue, which are summarized in this review. Strategies have been tested and developed, aiming to stratify the patients with a potential need for supplemental Se and how best to monitor Se supplementation with respect to health effects and risks. **Methods:** We analyzed blood and tissue Se-levels of different tumor patients (*n* = 512). Two randomized phase III clinical studies were conducted for testing a potential radioprotective effect of supplemental Se during radiation therapy in patients with uterine cancer (*n* = 81) and head and neck tumor patients (*n* = 39). **Results:** A relative Se deficit in whole blood or serum was detected in the majority of tumor patients (carcinomas of the uterus, head and neck, lung, rectal or prostate cancer). In prostate cancer, tissue Se concentrations were relatively elevated in the carcinoma centre as compared to the surrounding compartment or as compared to tumor samples from patients with benign prostatic hyperplasia. Adjuvant Se supplementation successfully corrected Se-deficiency in the patients analyzed and decreased radiotherapy-induced diarrhea in a randomized study of radiotherapy patients with carcinomas of the uterus. Survival data imply that Se supplementation did not interfere with radiation success. Some positive effects of supplemental Se in the prevention of ageusia (loss of taste) and dysphagia due to radiotherapy were noted in a second randomized trial in patients with head and neck cancer. We have not observed any adverse effects of supplemental Se in our studies. **Conclusions:** Se supplementation yielded promising results concerning radioprotection in tumor patients and should be considered as a promising adjuvant treatment option in subjects with a relative Se deficit.

## 1. Introduction (Why Should a German Radiation Oncologist Be Interested in Se)

Supplementation with the essential trace element Se is increasingly recognized as a promising adjuvant treatment option by radiation oncology expert groups. Cancer-preventive and cytoprotective activities of supplemental Se have been reported in a number of animal cancer models and clinical trials. A sufficiently high Se intake is needed to support the regular biosynthesis of selenoproteins, which are centrally implicated in redox regulation and antioxidant functions. By these and additional mechanisms, selenoproteins protect membrane integrity, contribute to regular energy metabolism and prevent DNA damage [1,2,3,4].

Compared to the USA, the population in many European countries including Germany needs to be considered as slightly Se deficient. Soil Se contents differ strongly around the world and therefore also the Se content of the food obtained from different soils reflects this difference [3,5]. Experimental evidence from preclinical and human studies indicate that a low Se status may augment radiation-induced damage while a sufficiently high Se supply might elicit protective activities and reduce the risk of side effects from the therapeutic radiation exposure (Table 1) [6,7,8,9,10,11,12,13,14,15,16].

For these and other reasons, the popularity of self-medication with supplemental Se is increasing in both healthy and diseased subjects, especially in cancer patients who hope to improving their quality of life, reducing side-effects during therapies, strengthening their immune system and supporting their recovery. However, such self-treatment is usually not reported to the treating oncologist, causing some degree of uncertainty during therapy [17].

For obvious reasons, the radiation oncologist is mainly interested in whether supplemental Se may affect therapeutic effects by either altering the sensitivity of healthy tissue to the damaging radiation or by even improving the curative effects of therapeutic radiation on the malignant tissue.

## 2. Se Measurement in Tumor Patients

The German Working Group Trace Elements and Electrolytes in Oncology (AKTE, Bielefeld, Germany) has performed several studies in which Se status were measured in a number of tumor patients (*n* = 512). The majority of patients with different tumors (head and neck, lung or rectal cancer, carcinomas of the uterus and prostate cancer, respectively) showed Se concentrations in whole blood that were below the reference range of 110 to 130 µg/L [18]. Interestingly, the tissue Se concentrations were elevated in the carcinoma as compared to the compartment surrounding it. Moreover, also tumor tissues from patients with benign prostatic hyperplasia displayed lower Se concentrations than prostate carcinoma tissue and circulating Se and selenoprotein P concentrations were lower in patients as compared to controls (Table 2) [19,20,21,22,23,24].

These results motivated us to test whether adjuvant Se supplementation may elicit beneficial effects in our tumour patients and provided the scientific rationale for our clinical analytical and intervention studies.

## 3. Clinical Studies on the Effects of Supplemental Se During Radiotherapy

The homeostasis of the human organism is challenged by radio- and chemotherapy and side effects are occurring. A Se-deficient cancer patient may be at specific risk for the unwanted consequences of therapy as usually the nutritional situation in the clinics is sub-optimal and the ongoing disease may impair regular trace element metabolism even further [25]. For these reasons, several studies have been conducted and indicated that supplemental Se elicits some protecting effects on the normal tissue thereby alleviating therapy-induced side effects [26,27,28,29,30,31].

Hence, we initiated the first randomized phase III clinical trials in radiation oncology to test whether adjuvant sodium selenite supplementation improves therapy success and protects normal tissue from damage thereby reducing side effects. The first study was conducted with gynecological patients who received supplemental Se after surgery during radiotherapy. The primary study aims were to verify that adjuvant supplementation successfully increased the Se status in patients and whether this improvement is associated with reduced radiation-induced diarrhea, as this complication constitutes a relevant and frequent side effect in patients treated by pelvic radiotherapy for uterine or cervical cancer. We were able to enroll 81 patients, 39 of them into the Se group (SeG) and 42 into the control group (CG). As expected, both whole blood and plasma Se levels increased in the SeG. Importantly, side effects were less frequent and severe in the SeG and the actuarial incidence of at least CTC 2 diarrhea was significantly reduced in the SeG as compared to the CG. The calculated 10-year overall survival rate of patients in the SeG was 55.3% compared to 42.7% in the CG (*p* = 0.09). The 10-year disease free survival rate was not different, i.e., a calculated 80.1% in Se-supplemented as compared to 83.2% in the control patients (*p* = 0.65). There was a tendency that the patients with higher Se status tolerated the radiotherapy better than the ones with relative Se deficiency. Notably, the long-term survival rates were similar between the groups. These observations support the notion that supplemental Se is safe and may indeed elicit some beneficial effects on normal tissue without conferring protection to tumor cells, in clear contrast to what was suspected and assumed by many colleagues [32,33].

A second randomized Se supplementation study in radiation therapy was conducted with patients suffering from head and neck tumors. This study was conducted with 39 patients, of which 22 were enrolled into the SeG and 17 as controls. A significant reduction of dysphagia during the last week of irradiation was observed in response to supplemental Se, especially in the patients with higher plasma and whole blood Se levels (*p* = 0.04). Similarly, also loss of taste (ageusia) as a typical side effect was observed more frequently in the CG as compared to the SeG, however without reaching statistical significance (*p* = 0.172) [34].

Collectively, there are scientific experiences with Se in radiotherapy worldwide from at least sixteen clinical studies conducted between 1987 and 2012. From all these study results, it can be concluded that adjuvant Se supplementation tends to improve the general conditions of the patients, their quality of life while at the same time reducing the side effects of radiotherapy. Selenium supplementation did not reduce the effectiveness of radiotherapy and no toxicities were observed under these conditions [35].

## 4. Discussion

The Se status of tumor patients tends to be reduced as compared to control subjects. Adjuvant Se supplementation improves radioprotection of healthy tissue in tumor patients. Nevertheless, our understanding of Se biology in radiation oncology is still very limited and additional clinical studies are needed in order to better appreciate this issue and enable respective adjuvant treatment options. Unfortunately, there are reports claiming that Se supplementation may lead to an increased risk of developing diabetes mellitus. These critical and mainly US-based studies highlighted again the narrow therapeutic width of Se intake in humans and the potential risk associated with Se supplementation of already well-supplied subjects [36,37]. And notably, both studies used Se-rich yeast and pure selenomethionine, respectively and should thus not be extrapolated to short term supplementation with inorganic selenite.

Nevertheless, in view of the marginal Se intake of many Europeans, the majority of patients is likely to benefit from supplemental Se as the potential side-effects have observed in already well-supplied subjects only and not in Se-deficient subjects. Assuming a U-shaped curve of Se status and health effects and in view of the marginal Se supply of Europeans as compared to e.g., US Americans, we conclude that in Europe there is little risk of over-supplementation of cancer patients when using the recommended dosages before and during radiation therapy [18,38,39,40].

Unfortunately, even nowadays there is not yet a general recommendation in favor of or against Se supplementation in cancer patients. Nevertheless, more and more patients treated with curative or palliative radiotherapy in Germany are using adjuvant Se supplementation in a self-motivated and uncontrolled manner. In the future, the practitioner-recommended as well as the self-chosen supplementations with Se need to be better monitored and their effects systematically analyzed. At present, we strongly advocate to take the Se status of tumor patients under oncological therapy and aftercare situation more seriously into account and to consider a respective supplementation before, during or after therapy when the current Se status appears insufficient. To this end, Se status needs to be measured and monitored before, during and after radiation therapy [18].

When we started our research on Se 15 years ago, the oncological community in Germany was very skeptical, since supplemental Se was considered rather toxic than health supporting. 15 years later, a solid data basis exists and some radiation oncologists measure even the Se levels during therapy and compensate in cases of deficiency. But we have to keep in mind that the trace element Se constitutes a small piece only in the bigger puzzle on the parameters affecting therapeutic success in radiation oncology. Still, first convincing results have been achieved and showed that it is worthwhile to conducting further basic and clinical research in this field. The notion that there are no indications that supplemental Se in recommended dosages harms when given to subjects with proven Se deficit provides some confidence into this adjuvant therapy support.

## 5. Conclusions

Se supplementation has yielded positive and promising results concerning radioprotection in tumor patients. It can thus be considered as a meaningful and promising adjuvant treatment option in subjects with a proven Se deficit. Future clinical studies are needed to better identify those patients who benefit most from supplemental Se, i.e., likely the ones with the lowest baseline Se status. Yet, this hypothesis needs to be tested in sufficiently large intervention studies, in order to better characterize the best supplementation regimen during therapy.

## Figures and Tables

**Table 1:** Experimental evidence from preclinical and human studies on Se-use as an radioprotector/radiosensitizer.

Effects in	Substances	Examined Criteria	Results	Author
Living rats	Seleno-cystine, seleno-methionine, colloidal selenium, seleno-xanthene, seleno-xanthone, and seleno-chromone	Survival, Leucocytes following exposition to irradiation of 600, 750, and 900 R	Alleviation of mortality and leucopenia	Breccia et al. [6]
CHO AA8 cells (chinese hamster)	Sodium selenite and aminothiol WR-1065	Mutation frequency following exposure to 8 Gy	Protection against radiation-induced mutation was observed for both sodium selenite or WR-1065	Diamond et al. [7]
Tumor cells and tissues	Sodium selenite	Investigation of effect on the tumor response following selenium supplementation (review)	No detection of increased tumor tolerance	Doerr [8]
Mouse oral mucosa	Sodium selenite subcuta-neously or locally	Early radiation mucositis following single-dose and fractionated irradiation	Significant positive effect during the initial treatment phase	Gehrisch et al. [9]
Constituent bone cells and Ewings sarcoma of bone and rhabdomyosarcoma	Amifostine and sodium selenite	Investigation of effects on radioprotection and tumor response	Significant radioprotection to constituent bone cells while not protecting the tumor cells, increased cytotoxicity in nonirradiated and irradiated tumor cells	Margulies et al. [10]
Rats	Sodium selenite and vitamin E	Radiation-induced intestinal injury	Selenium and/or vitamin E pretreatments ameliorated disturbances in prooxidant-antioxidant balance, this amelioriation has been verified with histopathological findings	Mutlu-Tuerkoglu et al. [11]
C3H/HeN mice	Amifostine, sodium selenite and glucan	Survival-enhancing and hemopoietic-regenerating effects following exposure to 60 Co radiation	All treatments increased numbers of hemopoietic stem cells, Combined modality treatments were more effective than single-agent treatments	Patchen et al. [12]
Normal human skin fibroblasts and squamous cell carcinoma cells	Sodium selenite	Quantitative cell culture analyses following single-dose (0 to 7 Gy) and multiple fractionated-dose (5 × 2 Gy) irradiation	Sodium selenite under both radiation exposure conditions positively modulates the radiation response of normal fibroblasts, on the contrary, human tumor cells are not affected by the radioprotective capacity	Rodemann HP et al. [13]
Human umbilical vein endothelial cells and tumor cells of the HeLa, MIA Paca-2 and SiHa cell line	Sodium selenite	Proliferative activity after single-dose irradiation with 2 or 10 Gy	Sodium selenite can counteract the decrease of proliferative activity caused by irradiation in human endothelial cells, this effect was observed by far stronger in endothelial cells than in tumor cells	Schleicher U et al. [14]
C6 rat glioma cells	Sodium selenite	Effect after fractionated irradiation	Radiosensitizing effect of selenite	Schueller P et al. [15]
Male CD2F1 mice	Sodium selenite	Post-irradiation survival	Se-injection alone (1.6 mg/kg) 24 h before cobalt-60 irradiation increased the survival significantly	Weiss JF et al. [16]

**Table 2:** Number and mean values of patients included in studies of our working group with measurement of Se and Se-PP.

Diagnoses	Parameter	Material	Number	Mean Level	Author
Non small cell lung cancer and rectal cancer	Selenium	Serum	*n* = 20	54.4 µg/L (SD = 18.5 µg/L)	Muecke et al. [19]
Prostate cancer	Selenium	Whole blood	*n* = 24	60.1 µg/L (SD = 17.2 µg/L)	Muecke et al. [20]
	Selenium	Tissue of benign prostate hyperplasia	*n* = 22	198 µg/L (SD = 92.3 µg/L)	
	Selenium	Benign tissue surrounding the cancer	*n* = 9	139 µg/L (SD = 61.5 µg/L)	
Uterine squamous cell cancer and uterine adenocarcinoma	Selenium	Serum	*n* = 126	62.9 µg/L (SD = 18.3 µg/L)	Muecke et al. [21]
Prostate cancer	Selenium	Serum	*n* = 90	81.4 µg/L (67.9–98.4)	Meyer et al. [22]
	Selenoprotein P	Serum		2.9 mg/L (1.1–5.5)	
Head and neck tumors and carcinomas of the uterus	Selenium	Serum	*n* = 121	59.2 µg/L (SD = 13.5 µg/L)	Buentzel et al. [23]
Head and neck tumors	Selenium	Serum	*n* = 100	60.6 µg/L (SD = 13.4 µg/L)	Buentzel et al. [24]

## References

[1] Micke O., Muecke R., Buentzel J., Kisters K., Schaefer U., Elements G.W.G.T. (2010). Some more steps on the way to clinical elementology. Trace Elem. Electrolytes.

[2] Papp L.V., Lu J., Holmgren A., Khanna K.K. (2007). From selenium to selenoproteins: Synthesis, identity, and their role in human health. Antioxid. Redox Signal..

[3] Rayman M.P. (2000). The importance of selenium to human health. Lancet.

[4] Schomburg L., Schweizer U., Kohrle J. (2004). Selenium and selenoproteins in mammals: Extraordinary, essential, enigmatic. Cell Mol. Life Sci..

[5] Rayman M.P. (2012). Selenium and human health. Lancet.

[6] Breccia A., Badiello R., Trenta A., Mattii M. (1969). On the chemical radioprotection by organic selenium compounds in vivo. Radiat. Res..

[7] Diamond A.M., Dale P., Murray J.L., Grdina D.J. (1996). The inhibition of radiation-induced mutagenesis by the combined effects of selenium and the aminothiol wr-1065. Mutat. Res..

[8] Dorr W. (2006). Effects of selenium on radiation responses of tumor cells and tissue. Strahlenther. Onkol..

[9] Gehrisch A., Dorr W. (2007). Effects of systemic or topical administration of sodium selenite on early radiation effects in mouse oral mucosa. Strahlenther. Onkol..

[10] Margulies B.S., Damron T.A., Allen M.J. (2008). The differential effects of the radioprotectant drugs amifostine and sodium selenite treatment in combination with radiation therapy on constituent bone cells, ewing’s sarcoma of bone tumor cells, and rhabdomyosarcoma tumor cells in vitro. J. Orthop. Res..

[11] Mutlu-Turkoglu U., Erbil Y., Oztezcan S., Olgac V., Toker G., Uysal M. (2000). The effect of selenium and/or vitamin e treatments on radiation-induced intestinal injury in rats. Life Sci..

[12] Patchen M.L., MacVittie T.J., Weiss J.F. (1990). Combined modality radioprotection: The use of glucan and selenium with wr-2721. Int. J. Radiat. Oncol. Biol. Phys..

[13] Rodemann H.P., Hehr T., Bamberg M. (1999). Relevance of the radioprotective effect of sodium selenite. Med. Klin. (Munich).

[14] Schleicher U.M., Lopez Cotarelo C., Andreopoulos D., Handt S., Ammon J. (1999). Radioprotection of human endothelial cells by sodium selenite. Med. Klin. (Munich).

[15] Schueller P., Puettmann S., Micke O., Senner V., Schaefer U., Willich N. (2004). Selenium influences the radiation sensitivity of c6 rat glioma cells. Anticancer Res..

[16] Weiss J.F., Hoover R.L., Kumar K.S. (1987). Selenium pretreatment enhances the radioprotective effect and reduces the lethal toxicity of wr-2721. Free Radic. Res. Commun..

[17] Micke O., Bruns F., Glatzel M., Schonekaes K., Micke P., Mucke R., Buntzel J. (2009). Predictive factors for the use of complementary and alternative medicine (CAM) in radiation oncology. Eur. J. Integr. Med..

[18] Muecke R., Schomburg L., Buentzel J., Kisters K., Micke O., Oncolo T.E.E. (2010). Selenium or no selenium-that is the question in tumor patients: A new controversy. Integr. Cancer Ther..

[19] Mucke R., Micke O., Schonekaes K.G. (2000). Serum selenium levels, glutathione peroxidase activities and serum redox potential levels in patients with untreated non-small cell lung cancer and adenocarcinoma of the rectum. Trace Elem. Electrolytes.

[20] Muecke R., Klotz T., Giedl J., Buentzel J., Kundt G., Kisters K., Prott F.J., Micke O. (2009). Whole blood selenium levels (WBSL) in patients with prostate cancer (PC), benign prostatic hyperplasia (BPH) and healthy male inhabitants (HMI) and prostatic tissue selenium levels (PTSL) in patients with PC and BPH. Acta Oncol..

[21] Muecke R., Buentzel J., Glatzel M., Bruns F., Kisters K., Prott F.J., Schmidberger H., Micke O. (2009). Postoperative serum and whole blood selenium levels in patients with squamous cell and adenocarcinomas of the uterus after curative surgical treatment. Trace Elem. Electrolytes.

[22] Meyer H.A., Hollenbach B., Stephan C., Endermann T., Morgenthaler N.G., Cammann H., Kohrle J., Jung K., Schomburg L. (2009). Reduced serum selenoprotein p concentrations in german prostate cancer patients. Cancer Epidemiol. Biomarkers Prev..

[23] Buntzel J., Micke O., Kisters K., Bruns F., Glatzel M., Schonekaes K., Kundt G., Schafer U., Mucke R. (2010). Selenium substitution during radiotherapy of solid tumours-laboratory data from two observation studies in gynaecological and head and neck cancer patients. Anticancer Res..

[24] Buntzel J., Knolle U., Garayev A., Mucke R., Schafer U., Kisters K., Schonekaes K.G., Hunger R., Bruns F., Glatzel M. (2010). Trace elements selenium and zinc as tumor markers in patients with advanced head and neck cancer. Trace Elem. Electrolytes.

[25] Muecke R., Micke O., Schomburg L., Buentzel J., Kisters K., Adamietz I.A., Elements G.W.G.T. (2011). Selenium in radiation oncology—Experiences and prospects. Trace Elem. Electrolytes.

[26] Asfour I.A., El Shazly S., Fayek M.H., Hegab H.M., Raouf S., Moussa M.A. (2006). Effect of high-dose sodium selenite therapy on polymorphonuclear leukocyte apoptosis in non-hodgkin’s lymphoma patients. Biol. Trace Elem. Res..

[27] Asfour I.A., Fayek M., Raouf S., Soliman M., Hegab H.M., El-Desoky H., Saleh R., Moussa M.A. (2007). The impact of high-dose sodium selenite therapy on bcl-2 expression in adult non-hodgkin’s lymphoma patients: Correlation with response and survival. Biol. Trace Elem. Res..

[28] Asfour I.A., El-Tehewi M.M., Ahmed M.H., Abdel-Sattar M.A., Moustafa N.N., Hegab H.M., Fathey O.M. (2009). High-dose sodium selenite can induce apoptosis of lymphoma cells in adult patients with non-hodgkin’s lymphoma. Biol. Trace Elem. Res..

[29] Hu Y.J., Chen Y., Zhang Y.Q., Zhou M.Z., Song X.M., Zhang B.Z., Luo L., Xu P.M., Zhao Y.N., Zhao Y.B. (1997). The protective role of selenium on the toxicity of cisplatin-contained chemotherapy regimen in cancer patients. Biol. Trace Elem. Res..

[30] Sieja K., Talerczyk M. (2004). Selenium as an element in the treatment of ovarian cancer in women receiving chemotherapy. Gynecol. Oncol..

[31] Weijl N.I., Elsendoorn T.J., Lentjes E.G., Hopman G.D., Wipkink-Bakker A., Zwinderman A.H., Cleton F.J., Osanto S. (2004). Supplementation with antioxidant micronutrients and chemotherapy-induced toxicity in cancer patients treated with cisplatin-based chemotherapy: A randomised, double-blind, placebo-controlled study. Eur. J. Cancer.

[32] Muecke R., Schomburg L., Glatzel M., Berndt-Skorka R., Baaske D., Reichl B., Buentzel J., Kundt G., Prott F.J., deVries A. (2010). Multicenter, phase 3 trial comparing selenium supplementation with observation in gynecologic radiation oncology. Int. J. Radiat. Oncol..

[33] Muecke R., Micke O., Schomburg L., Glatzel M., Reichl B., Kisters K., Schaefer U., Huebner J., Eich H.T., Fakhrian K. (2014). Multicenter, phase iii trial comparing selenium supplementation with observation in gynecologic radiation oncology: Follow-up analysis of the survival data 6 years after cessation of randomization. Integr. Cancer Ther..

[34] Buntzel J., Riesenbeck D., Glatzel M., Berndt-Skorka R., Riedel T., Mucke R., Kisters K., Schonekaes K.G., Schafer U., Bruns F. (2010). Limited effects of selenium substitution in the prevention of radiation-associated toxicities. Results of a randomized study in head and neck cancer patients. Anticancer Res..

[35] Puspitasari I.M., Abdulah R., Yamazaki C., Kameo S., Nakano T., Koyama H. (2014). Updates on clinical studies of selenium supplementation in radiotherapy. Radiat. Oncol..

[36] Lippman S.M., Klein E.A., Goodman P.J., Lucia M.S., Thompson I.M., Ford L.G., Parnes H.L., Minasian L.M., Gaziano J.M., Hartline J.A. (2009). Effect of selenium and vitamin e on risk of prostate cancer and other cancers: The selenium and vitamin e cancer prevention trial (select). JAMA.

[37] Stranges S., Marshall J.R., Natarajan R., Donahue R.P., Trevisan M., Combs G.F., Cappuccio F.P., Ceriello A., Reid M.E. (2007). Effects of long-term selenium supplementation on the incidence of type 2 diabetes: A randomized trial. Ann. Intern. Med..

[38] Micke O., Schomburg L., Kisters K., Buentzel J., Huebner J., Muecke R. (2013). Selenium and hypertension: Do we need to reconsider selenium supplementation in cancer patients?. J. Hypertens..

[39] Muecke R., Micke O., Schomburg L., Kisters K., Buentzel J., Huebner J., Kriz J. (2014). Selenium supplementation in radiotherapy patients: Do we need to measure selenium levels in serum or blood regularly prior radiotherapy?. Radiat. Oncol..

[40] Muecke R., Micke O., Schomburg L., Buentzel J., Adamietz I.A., Huebner J., Elements G.W.G.T. (2014). Serum selenium deficiency in patients with hematological malignancies: Is a supplementation study mandatory?. Acta Haematol..

